# Patient Satisfaction With Speech Recognition in the Exam Room: Exploratory Survey

**DOI:** 10.2196/42739

**Published:** 2023-04-05

**Authors:** Jeffrey Sippel, Tim Podhajsky, Chen-Tan Lin

**Affiliations:** 1 Department of Internal Medicine University of Colorado School of Medicine Aurora, CO United States

**Keywords:** speech recognition, exam room, primary care, general practitioner, satisfaction, survey, perception, opinion, speech, voice, eHealth, digital health, health technology, communication technology

## Abstract

**Background:**

Medical speech recognition technology uses a microphone and computer software to transcribe the spoken word into text and is not typically used in outpatient clinical exam rooms. Patient perceptions regarding speech recognition in the exam room (SRIER) are therefore unknown.

**Objective:**

This study aims to characterize patient perceptions of SRIER by administering a survey to consecutive patients scheduled for acute, chronic, and wellness care in three outpatient clinic sites.

**Methods:**

We used a microphone and medical speech recognition software to complete the “assessment and plan” portion of the after-visit summary in the patient’s presence, immediately printed the after-visit summary, and then administered a 4-question exploratory survey to 65 consecutive patients in internal medicine and pulmonary medicine clinics at an academic medical center and a community family practice clinic in 2021 to characterize patient perceptions of SRIER. All questions were completed by all participants.

**Results:**

When compared to patients’ recollection of usual care (visits with no microphone and an after-visit summary without an “assessment and plan”), 86% (n=56) of respondents agreed or strongly agreed that their provider addressed their concerns better, and 73% (n=48) agreed or strongly agreed that they understood their provider’s advice better. A total of 99% (n=64) of respondents agreed or strongly agreed that a printed after-visit summary including the “assessment and plan” was helpful. By comparing the “agree” and “strongly agree” responses to the neutral responses, we found that patients felt that clinicians using SRIER addressed their concerns better (*P*<.001), they understood their clinician’s advice better (*P*<.001), and receiving a paper summary was helpful (*P*<.001). Patients were likely to recommend a provider using a microphone based on the Net Promoter Score of 58.

**Conclusions:**

This survey suggests patients have a very positive perception of speech recognition use in the exam room.

## Introduction

Health care is increasingly complex due to rising patient severity of illness, electronic health record (EHR) and documentation requirements, and institutional demands to see more patients in a shorter amount of time [1,2]. Simultaneously, clinician burnout is growing due to increases in cognitive workload [1]. The COVID-19 pandemic has further accentuated clinician stress and burnout since 2020 [3]. Taken together, these factors work against The Quadruple Aim of health care, which acknowledges the need to improve the work life and professional fulfillment of clinicians [4].

Speech recognition software transcribes the spoken word into text by using a dedicated microphone connected to a computer in conjunction with speech recognition software. Speech recognition has been primarily used to enhance clinician documentation [5]. Although there was dissatisfaction with early versions secondary to time lags and transcription errors, the accuracy and performance of speech recognition have greatly improved [5]. Speech recognition continues to gain popularity in the medical field and has been favorably received by clinicians to improve EHR efficiency [6]. However, speech recognition is not typically used in the clinical exam room in outpatient settings. Patient perceptions regarding the use of speech recognition in the exam room (SRIER) are therefore unknown. If speech recognition were used simultaneously with the EHR in the exam room, a real-time transcription would be available for immediate review. Its use in the exam room to provide a summary of the clinical encounter, such as an “assessment and plan,” may be a patient satisfier by providing a reflective listening opportunity for both patient and clinician, and by documenting the care plan in real time. The transcription can also be printed in the after-visit summary and given to the patient. This report describes our efforts using SRIER and its impact on patient perceptions.

## Methods

### Study Setting and Participants

We conducted an exploratory survey to examine patient perceptions regarding SRIER. A convenience sample of sequential appointments for acute, chronic, and wellness care was included. We administered surveys to 65 consecutive patients in the fall of 2021. The surveyors included the three authors, and all were attendings who worked in different outpatient clinic settings (internal medicine and pulmonary medicine clinics at an academic medical center and community family practice). Surveys were not administered in inpatient or emergency department settings.

### Study Protocol

We used medical speech recognition software and microphone hardware (Dragon Medical One and Dragon Powermic III by Nuance, Burlington, MA) to complete the patient’s “assessment and plan” in their presence in the exam room. This electronically transcribed “assessment and plan” was included in the printed after-visit summary, which was given to all patients immediately upon completion. After the clinic visit, each physician handed a paper survey to the patient. Then the physician left the room. The patient remained in the room to complete the survey, which was collected by a medical assistant. We did not use the Nuance Dragon Ambient eXperience system.

### Outcome Measures

The survey was comprised of four questions and comments. We selected three questions that would reflect patient perceptions of SRIER, focusing on the perceived effectiveness of the encounter and the value of a printed after-visit summary including the transcribed “assessment and plan.” We used a 5-point Likert scale for these questions (strongly disagree, disagree, neutral, agree, strongly agree) [7,8]. The last question asked whether the patient would recommend SRIER to others by using the Net Promoter Score (NPS) and asked for any comments [9]. All outpatient exam rooms were equipped with computers and microphone hardware, so there were no operational costs for this survey. This was considered a quality improvement project in the direct care of patients.

### Ethical Considerations

The Colorado Investigational Review Board deemed this survey as quality improvement and thus exempt from full review.

### Statistical Analysis

One of the authors (JS) collated data from the paper survey results. Descriptive analysis was used to characterize the ordinal variables on the Likert scale questions [7,8]. Standard NPS descriptive analysis was used for the NPS question [9]. To test the null hypothesis (that all participants would be “neutral” to SRIER for the Likert scale questions), we used a 1-sample *t* test 2-tailed analysis using Analysis ToolPak in Excel (Microsoft Corporation).

## Results

All questions were answered by 100% (N=65) of patients (Tables 1-3). Free-text comments were completed by 15% (n=10) of patients. The mean age was 62.8 (SD 12.6) years, and 55% (n=35) were male.

We tested the null hypothesis (that all participants would be “neutral” to SRIER for the Likert scale questions) using a 1-sample *t* test 2-tailed analysis. By comparing the “agree” and “strongly agree” responses to the neutral responses, we found that patients felt that clinicians using SRIER addressed their concerns better (mean score 4.4 out of 5, SD 0.86; *t*_64_=12.97; *P*<.001), they understood their clinician’s advice better (mean 4.2 out of 5, SD 1.03; *t*_64_=9.74; *P*<.001), and receiving a paper summary that included the transcribed “assessment and plan” was helpful (mean 4.8 out of 5, SD 0.47; *t*_64_=30.17; *P*<.001).

**Table 1:** Patients' perceptions of speech recognition in the exam room compared to visits with no microphones.

	My provider addressed my concerns better (“Agree or Strongly Agree”), n (%)	I understand my provider’s advice better (“Agree or Strongly Agree”), n (%)
All sites (N=65)	56 (86)	48 (74)
Community family practice (n=9)	5 (56)	6 (67)
Academic internal medicine (n=31)	28 (90)	24 (77)
Academic pulmonary (n=25)	23 (92)	18 (72)

**Table 2:** “Did you find it helpful to get a paper printout with what your provider said today?”

	“Agree or Strongly Agree,” n (%)
All sites (N=65)	64 (98)
Community family practice (n=9)	9 (100)
Academic internal medicine (n=31)	31 (100)
Academic pulmonary (n=25)	24 (96)

**Table 3:** “How likely are you to recommend a provider using a microphone in the exam room to other patients?”

	NPS^a,b^
Total (N=65)	58
Community family practice (n=9)	11
Academic internal medicine (n=31)	65
Academic specialty (n=25)	68

^a^NPS: Net Promoter Score.

^b^NPS ranges from –100 to 100.

## Discussion

### Principal Findings

Patients prefer speech recognition by their physician in the exam room compared to their recollection of usual care. Usual care does not include the use of a microphone with speech recognition and does not include a live narrative summary of the visit or routine printing of the after-visit summary. The process of listening to the physician verbally summarize their visit, then receiving a copy of this in their printed after-visit summary was rated positively, leading to feeling heard and understanding medical advice better. This exploratory survey supports speech recognition use in the exam room and suggests that SRIER can enhance physician-patient communication. Representative free-text comments from patients included the following:

Super helpful with the recap and microphone, helps me to ask any question in case I forget something.

The microphone allowed me to hear and read a second summary of my issue and treatment.

We observed that the community family practice clinic, although still receiving positive scores for the questions “provider addressed my concerns better” and “I understand my provider’s advice better,” did have scores lower than the academic internal medicine and pulmonary medicine clinics. This may be due to the small sample size. The community family practice clinic also had shorter appointment times than the other clinics, which may have contributed to the difference (20 minutes vs 30 minutes for established patients, and 40 minutes vs 60 minutes for new patients).

### Comparison With Other Work

This is the first report of speech recognition use in the exam room that we are aware of. The physician’s workflow included verbally summarizing the patient’s concerns and stating the “assessment and plan” in real time in the patient’s presence. The physician’s statements were captured as part of the EHR by speech recognition and printed in the after-visit summary. This workflow allows the provider to attend to the patient rather than typing notes into the computer, which improves patient comprehension and physician understanding. Reflective listening is a technique where the clinician repeats some of the patient’s words to indicate understanding. The use of speech recognition allows such word repetition to be documented in the note and serve a similar purpose. Verbally stating the “assessment and plan” allows patients to ask clarifying questions and correct misunderstandings. Since this portion of the documentation is completed in real time, it may reduce “pajama time” and the risk of burnout from after-hours work [6]. The real-time workflow also improves documentation accuracy by not relying on memory recall to complete notes hours or days later and reduces duplicate documentation. The printed summary reduces the risk of patients forgetting unwritten advice, which can be as high as 40%, and allows them to share advice with others [10]. These findings are relevant to acute, chronic, and wellness visits, and were not assessed in either the inpatient or emergency department settings.

### Limitations

There are several limitations to this exploratory survey. The project was conducted by three physicians at one health care organization with a limited set of patients, so the findings are not intended to be generalizable. The survey was conducted in outpatient clinic exam rooms, not in the inpatient or emergency department settings. Survey questions were not validated. Physician satisfaction was not assessed due to the small number of participants. There was not a control group for this survey, since the questions asked the patients to reflect and compare the current visit using speech recognition to their prior clinic visit experiences without speech recognition, essentially providing their own control. The accuracy of patient recall for satisfaction with prior visits was not validated.

Future investigation should expand on both patient and clinician experience with speech recognition in exam rooms. We are aware that developing technologies may capture full conversations between clinician and patient to auto-generate progress notes. It is clear that patients may be ready for such automation tools based on our initial findings.

### Conclusion

Patients have a very positive perception of speech recognition when used in the exam room. Periodic assessments such as this will be helpful to understand patient perceptions more fully as the use of technology by clinicians continues to change and expand. As speech recognition technology improves, similar surveys of patients and clinicians can guide the optimal use of such tools to improve communication, improve care, and reduce documentation burden.

## Abbreviations

EHR: electronic health record
NPS: Net Promoter Score
SRIER: speech recognition in the exam room
